# Are Incidental Gallbladder Cancers Missed with a Selective Approach of Gallbladder Histology at Cholecystectomy?

**DOI:** 10.1007/s00268-017-4215-0

**Published:** 2017-09-12

**Authors:** Linda Lundgren, Carolina Muszynska, Axel Ros, Gunnar Persson, Oliver Gimm, Lars Valter, Bodil Andersson, Per Sandström

**Affiliations:** 1 Department of Surgery, County Council of Östergötland, University Hospital of Linköping, SE-581 85 Linköping, Sweden; 2 Department of Clinical and Experimental Medicine, Faculty of Health Sciences, Linköping University, Linköping, Sweden; 3 Department of Clinical Sciences Lund, Surgery, Skåne University Hospital, Lund University, Lund, Sweden; 4 Department of Surgery, Ryhov Hospital, Jönköping, Sweden; 5 Department of Surgery, Eksjö Hospital, Eksjö, Sweden; 6 Research and Development Unit in Local Health Care, Linköping University, Linköping, Sweden

## Abstract

**Background:**

Incidental gallbladder cancer (IGBC) is an unexpected finding when a cholecystectomy is performed upon a benign indication, and the use of routine or selective histological analysis of gallbladder specimen is still debated. The aim of this study was to investigate whether the proportion of submitted gallbladder specimens for pathological investigation influences the proportion of IGBC found, and what possible factors preoperatively or perioperatively could influence the selection process.

**Methods:**

All cholecystectomies between January 2007 and September 2014 registered in the Swedish Registry of Gallstone Surgery and ERCP (GallRiks) were included. Proportion of histological analysis was divided into four subgroups (0–25%, >25–50%, >50–75%, >75–100%).

**Results:**

A total of 81,349 cholecystectomies were registered, and 36,010 (44.3%) gallbladder specimens were sent for histological analysis. A total of 213 cases of IGBC were discovered, which constituted 0.26% of all cholecystectomies performed and 0.59% of the number of gallbladder specimens sent for histological analysis. Hospitals submitting >75–100% of the gallbladder specimens had significantly more IGBC/1000 cholecystectomies performed (*p* = 0.003). Hospitals with the most selective approach had a significantly higher proportion of IGBC/1000 gallbladders that were sent for histological analysis (*p* < 0.001). Factors such as higher age (*p* < 0.001), female gender (*p* = 0.048) and macroscopic cholecystitis (*p* < 0.001) were more common in gallbladder specimens from hospitals that had a selective approach to histological analysis.

**Conclusion:**

A routine approach to histological analysis in cholecystectomies with a benign indication for surgery can uncover a higher proportion of IGBC cases. When a selective approach is used, risk factors should be taken into account.

## Introduction

Cholecystectomy is a common surgical operation, and approximately 11,000 cholecystectomies are performed annually in Sweden. In these cases, not all gallbladders are sent for histological analysis. The indications are not standardized and guidelines are missing, even though the main purpose of histology is to uncover incidental gallbladder cancer (IGBC).

The most common risk factors for gallbladder cancer consist of increased age, gallstone disease, female gender, infections (most commonly Salmonella) and chronic inflammation [1]. An elevated risk to find IGBC has been described for patients where laparoscopic surgery was converted to open surgery, and also for patients where alkaline phosphate levels were elevated before surgery [2].

Gallbladder cancer generally has a poor prognosis, and survival is undoubtedly dependent on an early diagnosis. The 5-year overall survival rate is 5% compared with a 79% survival rate when the disease is detected in its early stages [3, 4]. IGBC is a rare event and is present in 0.2–2.9% of cholecystectomies that are performed to treat benign gallstone disease [5, 6]. It represents 27–72% of all newly diagnosed gallbladder tumours [5, 7]. When gallbladder cancer is discovered unexpectedly, overall survival is increased if the patient undergoes a second extended surgery [8, 9].

When a histological analysis of the gallbladder specimen is routinely performed, all incidental gallbladder cancers can be expected to be found. Since histological analysis of the gallbladder specimen is not mandatory in Sweden, cases of IGBC can be missed.

There has, however, been a debate in the literature concerning the routine or selective histological assessment of a gallbladder specimen when a cholecystectomy is performed for benign causes. Support for routine assessment is based on the findings that also subclinical malignancies could be identified and that IGBC usually results in follow-up treatment [7, 10, 11]. The selective approach [2, 12–16], on the other hand, is supported by the fact that gallbladder cancer is unlikely to occur in a normal-looking gallbladder. In addition, the extra cost and time required to process the gallbladder specimen are arguments for selective assessment.

The aim of this study is to examine whether the proportion of histologically analysed gallbladder specimens that were collected in Swedish hospitals influenced the proportion of incidental gallbladder cancer cases that were detected, and whether there were any obvious preoperative or perioperative factors for the selection of specimens to be sent for analysis when a selective strategy for histological analysis was utilized.

## Materials and methods

This study was based on the Swedish Registry of Gallstone Surgery and ERCP (GallRiks). The registry was established in 2005. Since January 2007, it has also included information on whether the gallbladder specimen underwent histological analysis together with a general histological report. The registry is national and includes more than 90% of the cholecystectomies that are performed in Sweden and is continuously validated [17, 18]. The registry is web-based and consists of standardized variables.

All registered cholecystectomies between January 2007 and September 2014 from hospitals that registered more than 300 cholecystectomies (68 hospitals in total) were included in the study (*n* = 84,745); duplicates were excluded. Eleven hospitals were excluded that had registered 2–287 cholecystectomies during the study period. All of the patients with indications for surgery regarding a suspected malignancy and polyp(s) or indications that were subordinate to another abdominal surgery (*n* = 3396) were also excluded, leaving a cohort of 81,349 patients who underwent surgery for benign gallstone disease. To ensure that all patients with IGBC were included, the cohort with the histology report “cancer” (*n* = 224) in GallRiks and the cohort with missing data regarding the histology report (*n* = 3413) were cross-linked to additional registers as follows. For each patient in GallRiks, the unique Swedish personal identity number [19] was registered and cross-linkage to the National Board of Health and Welfare’s Cancer Register (www.socialstyrelsen; until December 2013) and the Swedish Registry for Cancer in the biliary tract and liver (SweLiv, www.cancercentrum.se/vast/cancerdiagnoser/lever-och-galla/kvalitetsregister; from June 2008).

**Fig. 1:**
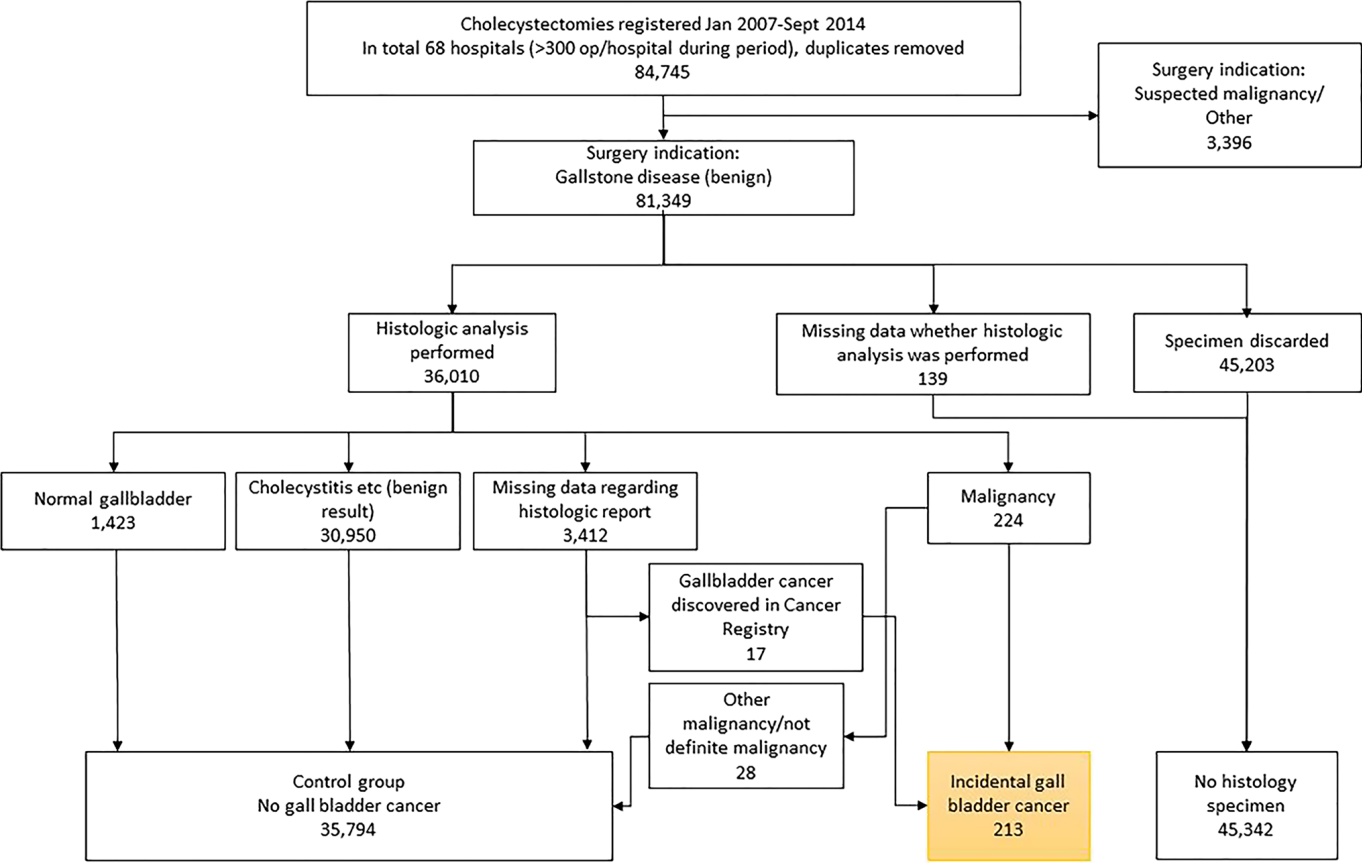
Flow chart for the selection process of the different groups that were included in the analysis based on the Swedish Registry of Gallstone Surgery and ERCP (GallRiks). Missing data were cross-linked with the Swedish Cancer Registry

The cross-linkage made it possible to retrieve TNM classification for IGBC patients, and if it was missing, the pathology report was collected from the actual hospital, which made it possible to find and include another 17 cases of IGBC and to exclude 28 cases without definite gallbladder cancer. The flow chart for the selection process is shown in Fig. 1.

All included cholecystectomies were divided into three cohorts: no histology (*n* = 45,339; 55.7%), histology without IGBC (*n* = 35,797; 44%) and histology with IGBC (*n* = 213; 0.26%). The cohorts are analysed according to variables in Tables 1 and 2.

**Table 1:** Preoperative data

	No histology *n* = 45,339	Histology without IGBC *n* = 35,797	IGBC *n* = 213	*p* value
Preoperative parameters age, (years)*	48 (±16)	55 (±16)	70 (±11)	<0.05
Gender				<0.05
Male	12,332 (27)	14,323 (40)	44 (21)	
Female	32,992 (73)	21,462 (60)	169 (79)	
Missing data**	15 (0)	12 (0)	0 (0)	
ASA				<0.05
1	25,634 (57)	16,416 (46)	50 (24)	
2	16,830 (37)	16,088 (45)	118 (55)	
3	2784 (6.1)	3139 (8.8)	41 (19)	
4**	80 (0.2)	145 (0.4)	4 (1.9)	
5**	11 (0)	9 (0)	0 (0)	
Jaundice***				<0.05
Yes	2974 (6.6)	3503 (9.8)	35 (16)	
No	42,365 (93)	32,294 (90)	178 (84)	
Indication for surgery				<0.05
Biliary colic	31,124 (69)	16,488 (46)	67 (32)	
Complication (cholecystitis, pancreatitis)	14,079 (31)	18,927 (53)	144 (68)	
Acalculous cholecystitis**	136 (0.3)	382 (1.1)	2 (0.9)	
Type of surgery				<0.05
Acute	11,637 (26)	13,771 (39)	105 (49)	
Elective	23,589 (74)	21,753 (61)	107 (50)	
Vital indication**	273 (0.8)	273 (0.8)	1 (0.5)	

Comparison of the three subgroups: gallbladders not submitted for histology (no histology), histology without IGBC (incidental gallbladder cancer) and IGBC

Values in parenthesis are percentages unless indicated otherwise

* Values are the mean (SD). ** Numbers not included in statistical analysis due to insufficient number. *** Bilirubin > 50 µmol/L and/or known bile duct stone

**Table 2:** Perioperative data

	No histology *n* = 45,339	Histology without IGBC *n* = 35,797	IGBC *n* = 213	*p* value
Perioperative data surgical procedure				<0.05
Laparoscopic	40,761 (90)	27,807 (78)	98 (46)	
Laparoscopic converted to open	1629 (3.6)	4250 (12)	60 (28)	
Open	2102 (4.6)	3303 (9.2)	53 (25)	
Subtotal**	27 (0.1)	43 (0.1)	1 (0.5)	
Minimal incision right subcostal**	820 (1.8)	394 (1.1)	1 (0.5)	
Reason for laparoscopic procedure converted to open				<0.05
Advanced cholecystitis	488 (1.1)	2014 (5.6)	26 (12)	
Intraabdominal adhesions	344 (0.8)	700 (2.0)	6 (2.8)	
Anatomy unclear	336 (0.7)	859 (2.4)	19 (8.9)	
Other	129 (0.3)	216 (0.6)	5 (2.3)	
Bleeding**	94 (0.2)	167 (0.5)	1 (0.5)	
Damage to extrahepatic bile ducts/bile duct gallstone**	238 (5.2)	294 (6.5)	3 (1.4)	
Not converted**	43,710 (96)	31,547 (88)	153 (72)	
Macroscopic assessment				<0.05
Normal gallbladder	32,133 (71)	10,078 (28)	13 (6.1)	
Acute/chronic cholecystitis	12,681 (28)	24,138 (67)	129 (61)	
Suspect malignancy or polyp	15 (0)	489 (1.3)	60 (28)	
Perforated gallbladder + other finding	510 (1.1)	1092 (3.1)	11 (5.2)	

Comparison of the three subgroups: gallbladders not submitted for histology (no histology), histology without IGBC (incidental gallbladder cancer) and IGBC

Values in parenthesis are percentages unless otherwise indicated

** Numbers not included in the statistical analysis due to an insufficient number

The hospitals were divided into four groups according to the proportion of gallbladder specimens that were submitted for histological analysis: 0–25%, >25–50%, >50–75% and >75–100%. The patients from each group of hospitals were compared regarding the proportion of IGBC/1000 cholecystectomies performed and IGBC/1000 cholecystectomies with histological analysis.

Data from each hospital were compared regarding mean age, proportion of females, macroscopic cholecystitis, laparoscopic surgery converted to open surgery, acute surgery and ongoing jaundice among patients whose gallbladder specimen was sent for histological analysis.

The outcome of the study was to investigate whether the proportion of gallbladders that were sent for histology from a specific hospital affected the number of cases of IGBC that were found. This was calculated as the proportion of IGBC/1000 cholecystectomies in total and IGBC/1000 cholecystectomies with histological analysis. Any differences in pre- and perioperative factors in patients from hospitals that had submitted a low or high proportion of gallbladder specimens were also analysed.

This study was approved by the Regional Ethical Committee in Linköping, Sweden (Dnr 2014/39-31).

## Statistical analysis

Statistical analyses were performed using the IBM^®^ SPSS^®^ Statistics software for Windows, Version 23.0, 2015 (IBM Corp, Armonk, NY, USA). Means and standard deviation were used for continuous variables. One-way ANOVA was used for continuous variables, and pairwise comparisons were made with Tukey’s post hoc test. Categorical data were compared using the Chi-square test. Linear regression was used to compare aggregated data for hospitals. The mean proportions of samples that were submitted for histological analysis in each of the four subgroups were compared using the Chi-square test. In the statistical analysis using linear regression, the frequency of IGBC from each hospital was counted separately and compared to one another; therefore, hospitals that had performed less than 300 cholecystectomies during the study period were excluded due to the low incidence of the disease. A *p* value of <0.05 was considered statistically significant.

## Results

### Comparison of the three subgroups: no histology, histology without IGBC and histology with IGBC

The cohort consisted of 81,349 patients with a mean age of 51 years, and 67% were women.

Pre- and perioperative data for the three subgroups are listed in Tables 1 and 2. IGBC patients were older, had a higher percentage of women and showed the highest proportion of American Society of Anesthesiologists (ASA) scores of 3. A higher percentage of patients with IGBC also were diagnosed with ongoing jaundice at the time of surgery and were more often operated on due to acute indications. A total of 68% of the IGBC cohort was operated on because of the presence of a gallstone complication, compared to 31% for the cohort without histological analysis. There was a higher proportion of cases in which a laparoscopic procedure had been converted to an open procedure in the IGBC cohort.

### Pathology reports from the gallbladders that were sent for histology

A total of 36,010 (44%) gallbladder specimens were sent for histological analysis, as shown in Fig. 1. The gallbladder specimen was diagnosed as normal by the pathologist in 1423 cases (4.0%) and as mixed benign pathology in 30,950 cases (86%), where acute and chronic cholecystitis accounted for the most in 28,514 cases (79%). A total of 213 cases of IGBC were confirmed, which rendered a frequency of 0.26% IGBC in all of the cholecystectomies that were performed and a frequency of 0.59% of all the gallbladder specimens that were sent for histological analysis.

### Perioperative macroscopic assessments and pathology reports for IGBC

**Fig. 2:**
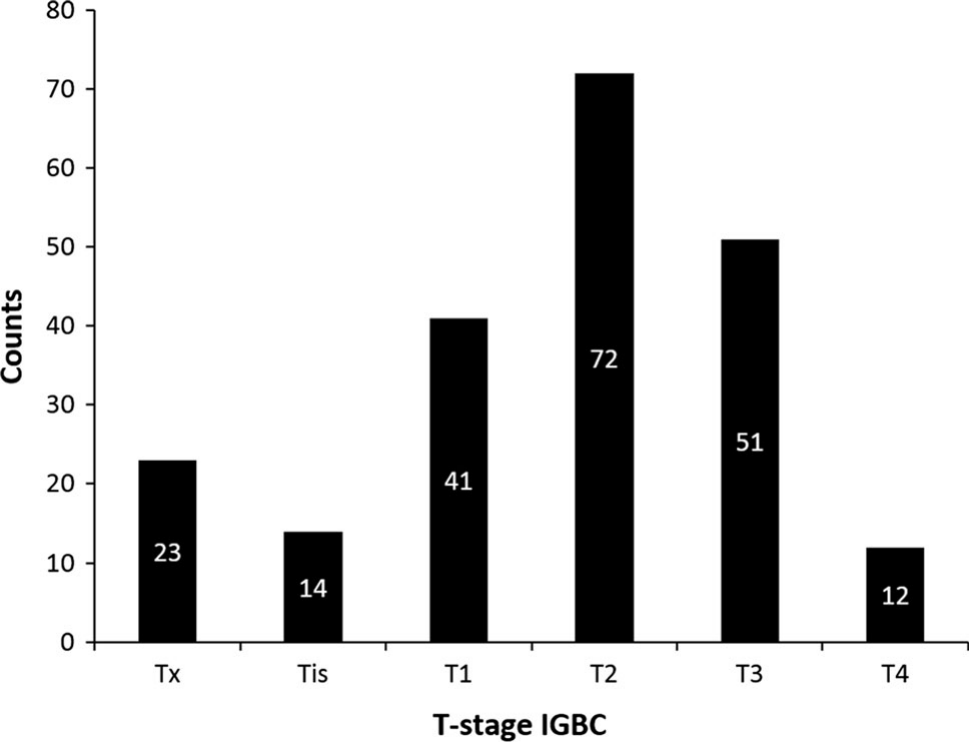
Distribution of T-stages for the IGBC (incidental gallbladder cancer) cohort (*n* = 213)

The macroscopic perioperative assessments of the 213 IGBC are described in Table 2. Suspected malignancy or polyp was registered in 60 cases (28%) and a normal gallbladder in 13 cases (6.1%). The complete distribution of T-stages for the IGBC cohort is shown in Fig. 2. The distribution of T-stages in the 13 specimens that were registered as normal was as follows: 2 Tx, 4 Tis, 1 T1, 5 T2 and 1 T3. In the IGBC cohort gallbladders with visible polyps or with macroscopically suspected malignancy, the distribution of the T-stages was: 8 Tx, 1 Tis, 7 T1, 18 T2, 20 T3 and 6 T4 with a seemingly larger amount of more advanced T-stages.

### Comparison between hospitals regarding proportion of histology and number of IGBC found

**Fig. 3:**
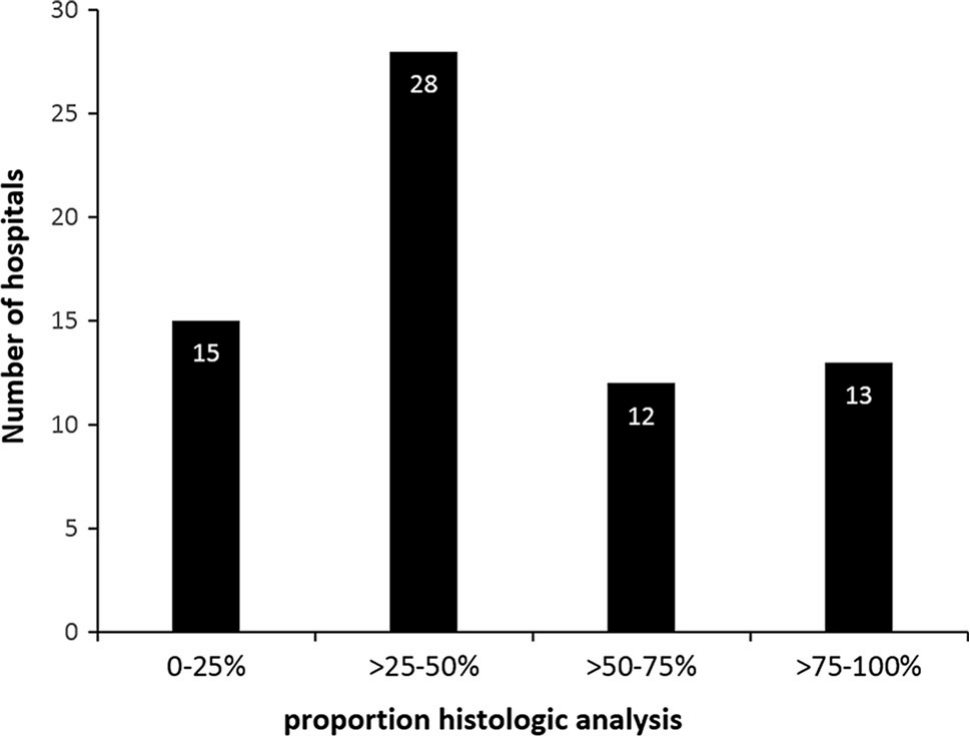
Sixty-eight hospitals that were included in the analysis were subdivided into four cohorts based on the proportions of gallbladders that were sent for histological analysis out of all cholecystectomies that were performed: 0–25%, >25–50%, >50–75%, >75–100%. Number of hospitals per subgroup

**Fig. 4:**
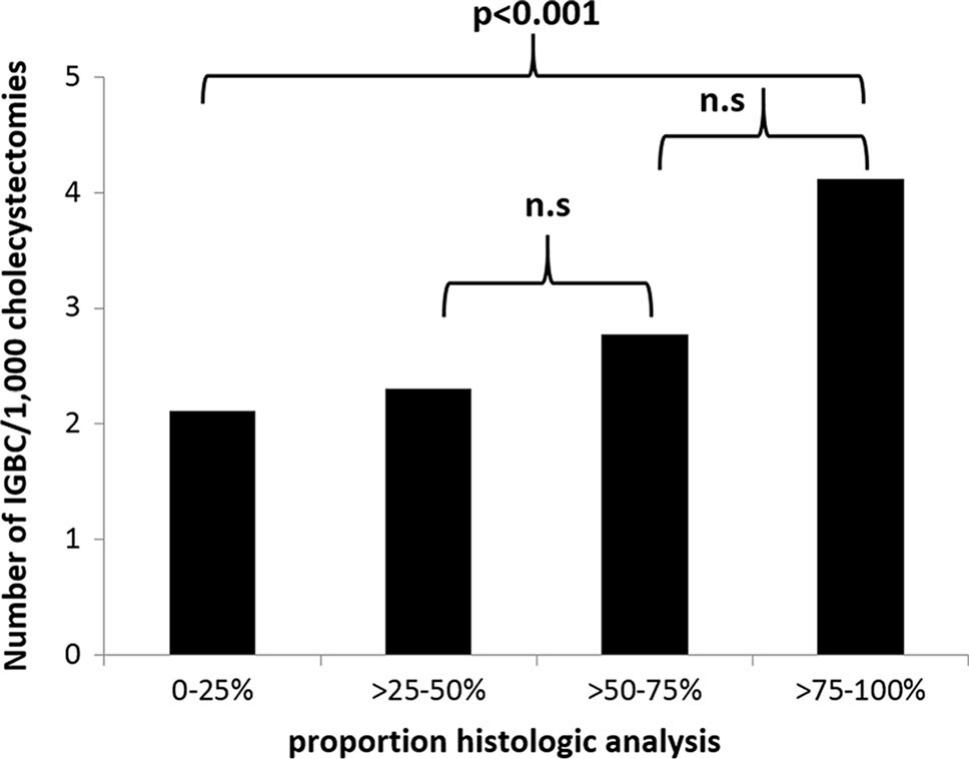
Sixty-eight hospitals that were included in the analysis were divided into in four groups based on the proportions of gallbladders that were sent for histological analysis out of all cholecystectomies that were performed: 0–25%, >25–50%, >50–75%, >75–100%. Hospitals that submitted a high proportion of gallbladder specimens found a higher proportion of incidental gallbladder cancer (IGBC) cases per cholecystectomy. Number of IGBC/1000 cholecystectomies performed in the four subgroups. n.s. = not significant

The proportion of submitting the gallbladder specimen was divided into four groups. The amount of hospitals for each group was distributed as follows: 0–25% 15, >25–50% 28, >50–75% 12, >75–100% 13 as shown in Fig. 3. Most hospitals analysed >25–50% of resected gallbladders. The number of IGBC/1000 cholecystectomies in relation to each subgroup is shown in Fig. 4. The hospitals in the subgroup of >75–100% found significantly more patients with IGBC than the other three groups (*p* = 0.003 between all four groups, *p* < 0.001 between subgroups >75–100% and 0–25%). This was also analysed for each hospital, showing the same results (*p* = 0.015).

**Fig. 5:**
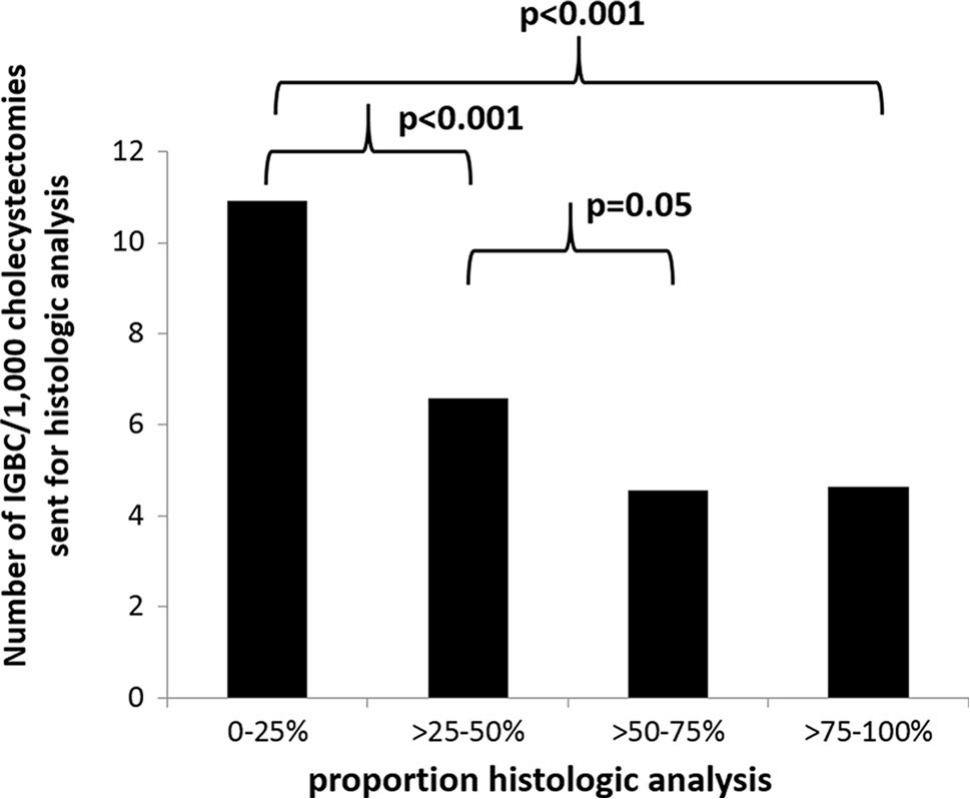
Sixty-eight hospitals that were included in the analysis were subdivided into four cohorts based on the proportions of gallbladders that were sent for histological analysis out of all of the cholecystectomies that were performed: 0–25%, >25–50%, >50–75%, >75–100%. Hospitals that submitted few gallbladder specimens (with a selective approach) found a higher proportion of incidental gallbladder cancer cases per cholecystectomy that were sent for histological analysis

If the proportion of IGBC cases was compared only among gallbladders from cholecystectomies that underwent histological analysis, the opposite relationship was found. The subgroup with the most selective approach (0–25%) found significantly more IGBC/1000 gallbladders that were sent for histological analysis than the other three groups (*p* < 0.001 between all groups, *p* < 0.001 between the 0–25% and >75–100% subgroups), as shown in Fig. 5. Using linear regression, comparing each hospital to one another, showed the same result (*p* = 0.005).

### Selection process for gallbladder specimens

Pre- and perioperative variables were analysed in cases where the gallbladder specimen was submitted. The hospitals with the most selective approach had a higher mean age of patients (*p* < 0.001) and the highest proportion of samples from women (*p* = 0.005). The proportion of macroscopically cholecystitis in the submitted specimens was also higher in hospitals with a selective approach (*p* < 0.001), as well as conversion of the laparoscopic procedure to an open procedure (*p* = 0.005) compared to the hospitals that had a routine approach for sending the gallbladder for histological analysis.

No differences could be found between hospitals that submitted many or few gallbladder specimens in terms of acute surgery and ongoing jaundice.

## Discussion

This study on a Swedish national prospective registry, GallRiks, was conducted to evaluate whether a selective approach in sending presumed benign gallbladders for histological analysis had an effect on the detection of incidental gallbladder cancer.

Our main finding was that hospitals that routinely submitted gallbladder specimens found a higher proportion of IGBC per cholecystectomy, compared to hospitals with a selective approach, indicating that a selective approach misses some of the IGBC cases and that macroscopic assessment of the gallbladder specimen is not enough to rule out gallbladder cancer. When the proportion of IGBC cases in cholecystectomies where the specimen was sent for histological analysis was evaluated, the hospitals with a selective approach found the highest proportion of IGBC cases, indicating that the selective process is not random. In the hospitals that used a selective approach, the gallbladder specimens that were submitted for histological analysis came from older patients, more often from female patients and showed a higher proportion of macroscopic cholecystitis. This indicates that the selection process is presumably based on the risk factors for gallbladder cancer, but it does not appear to be sufficiently accurate since some gallbladder cancers appeared to be overlooked. In a recent study presented by Muszynska et al. [20], multivariable analysis shows that IGBC is more common in women, in older patients and in patients with previous cholecystitis. However, it should be taken into account that if only risk factors provide the basis for selecting which gallbladder specimen to submit, there is a risk that early cancers in younger patients can be missed.

The proportion of IGBC cases in this study was 0.26% of all cholecystectomies and is consistent with previously published data in similar populations [2, 21–23]. In a recent published meta-analysis of 26 studies concerning IGBC, ten publications reported the incidence of IGBC, and in total, 403 cases of IGBC were detected in the 80,228 cholecystectomies; the pooled proportion of IGBC cases among the cholecystectomies performed for benign gallbladder disease was 0.7% [24]. It was not specified if the incidence was calculated based on the total number of cholecystectomies that were performed (routine approach) or if it was based on the number of cholecystectomies in which the gallbladder specimen was sent for histological analysis (selective approach). When assessed in relation to the cholecystectomies with completed histological analysis in our study, the proportion of IGBC cases was 0.59%.

There have been some controversies in the literature regarding whether all IGBCs have macroscopic abnormalities. For example, in the study from Agarwal et al. [11], 4 out of 470 gallbladder specimens had IGBC despite any suspicion of malignancy when the cut-open gallbladder specimen was examined. Others have found that all cases of IGBC show some abnormalities when assessed [2, 23]. Mittal et al. [25] examined 1312 patients who underwent cholecystectomy for gallstone disease; carcinoma was detected in 13 patients, and all of the specimens had some abnormalities. In the normal-looking gallbladders, no cancer was diagnosed. In our study, all except for 6.1% of the specimens in the IGBC cohort were abnormal in some way when they were macroscopically examined. Almost one-third of the specimens was suspected of being malignant or had polyps, and 60% were registered as macroscopic cholecystitis with a presumably wall-thickened gallbladder. Among the 13 specimens (6.1%) that were registered as macroscopically normal but were diagnosed to be cancer, the macroscopic evaluation can be questioned in at least 6 cases since the T-stage classifications in those samples were 5 cases with pT2 and one case with 1 pT3.

A hypothetical calculation may be obtained from this material. The group that sent >75–100% of the gallbladders for histological analysis found 4.1 cases of IGBC in 1000 samples from cholecystectomies. Using this number on the amount of all 81,349 cholecystectomies that were performed, one may estimate that there would have been 334 cases of IGBC in this material; therefore, 121 cases were overlooked. Unfortunately we do not have any way to control this number.

The awareness of risk factors for gallbladder cancer and a thorough inspection of the gallbladder specimen, including an opening of the specimen for a careful examination of the mucosa, could hopefully improve the frequency of IGBC discovery when a selective histological analysis for cholecystectomies that were performed with a benign indication is being used. The review presented by Jayasundara et al. [26] concludes that a selective approach for histological assessment could be an option in areas of very low incidence of gallbladder cancer, but universal selective histological analysis of gallbladder specimen is not to be recommended.

This study is limited by factors that are associated with an analysis of registry data. Also health economics should be taken into account.

The strength of this study is the nationwide population-based gallstone registry and cross-linkage to other registries increases the probability that we have covered all cases of IGBC during the study period.

## Conclusion

We have found that a routine approach of histological analysis in cholecystectomies that were performed in response to a benign indication can uncover a higher proportion of IGBC cases compared to a selective approach of histological analysis. Studies on patient outcome and health economics may further strengthen the recommendation for routine analysis of all gallbladder specimens. When using a selective approach, it is important to take into account the risk factors for gallbladder cancer and to carefully examine the gallbladder specimen before making the decision of whether to submit the specimen.
